# Predicting a Child's Oral Health Status from the Mother's Oral Health Behavior

**DOI:** 10.1055/s-0042-1757569

**Published:** 2022-12-13

**Authors:** Afra Fahira Rissetyo Utomo, Aulia Iskandarsyah, Arlette Suzy Setiawan

**Affiliations:** 1 Dental Undergraduate Study Program, Faculty of Dentistry, Universitas Padjadjaran, Indonesia; 2 Department Medical Psychology, Faculty of Psychology, Universitas Padjadjaran, Indonesia; 3 Department of Pediatric Dentistry, Faculty of Dentistry, Universitas Padjadjaran, Indonesia

**Keywords:** oral health behavior, oral health status, mother, child

## Abstract

**Objective** A mother has a decisive role in maintaining children's oral health, especially before the child is of preschool age. The behavior of mother becomes a source of learning for children who can determine the child's condition, including health behavior. This study analyzes the relationship between maternal oral health behavior and children's oral health status.

**Materials and Methods** The research methodology used is a quantitative observational study with a cross-sectional approach to determine the oral health behavior of mothers and children's oral health status. The population of this study were mothers with their first child aged between 3 and 5 years in Tanjungsari, Tanjungsari, Sumedang, West Java, Indonesia. The sampling method and technique used nonrandom and consecutive sampling from six health center units which yielded 46 mothers. Correlation analysis was done with chi-squared validity statistical test and Spearman rank correlation.

**Results** The score for the mother's oral health behavior was 75.54, while the score for the child's oral health status was 54.46. The results of the Spearman rank correlation test showed that the maternal oral health behavior score's correlation coefficient ( *r* ) was 0.198 ( *p* -value: 0.188). The calculation of the Spearman rank correlation shows that the mother's oral health behavior affects the child's oral health status in the food and beverage consumption selection.

**Conclusion** Based on all indicators of maternal oral health behavior studied, the behavior in maternal food and beverage consumption has a relationship with the child's oral health status, namely the better the behavior in the mother's food and drink consumption, the better the child's oral health status. Other indicators of oral health behavior did not show any relationship with the oral health status of children.

## Introduction

Families play an essential role in child development, especially mothers who have more contact with children daily. Mothers are the primary health care providers and role models for children, either directly by teaching their children or indirectly by imitating their parents. Mothers become one unit with children because mothers are members of a good health team to monitor children's health. In maintaining dental and oral health, parents must guide, provide understanding, remind, motivate, and provide facilities for children to maintain healthy teeth and mouth. 1 Oral health habits can affect oral health and the general health of each individual. 1 2 3 Application of the practice of maintaining oral health should be carried out from an early age by parents for their children, especially mothers because the application can shape children's character during the preschool period. 4

Ajzen explained that three determinants of psychological factors generally affect behavior in the theory of planned behavior. The determinants are attitudes, subjective norms, and behavioral perception control. 5 Behavior is seen from what parents feel and realize about their child's dental health. Parents' oral health behavior can affect children, such as the habit of determining the type of food, how to brush teeth, and use of toothpaste. Teeth contain fluoride and assess the choice of type of treatment when a child has dental and oral health problems. 1 2 Factors influencing oral health behavior are knowledge, attitudes, beliefs, values, behavior, socioeconomic status, availability of health facilities and facilities, or influenced by family, teachers, friends, and community leaders. 4

The developmental phase of children aged between 2 and 5 is known as the preschool phase. They have better motor skill development characteristics than in the previous phase; children can control and care for themselves. 6 Oral health habits and parental psychosocial factors can harm children's oral health, such as depression, low maternal coherence, and poor parenting. There is a relationship between parents' attitudes in the prevention of dental caries in children, and parents' attitude is proven to influence every action taken in educating children regarding dental care. There is a significant relationship between a mother's knowledge about brushing with the behavior and knowledge of mothers in educating children to brush their teeth has been produced in several studies. 7

Appropriate oral health behavior will result in a child's good oral health status. Vice versa, inappropriate oral health behavior applied to children can affect the oral health behavior that children use in their daily lives so that it will affect children's oral health conditions both in quality and quantity. 7 Based on Indonesian Basic Health Research conducted in 2018 in West Java Province, the proportion of oral health diseases in children aged between 3 and 4 years and 5 and 9 years was 8.8 and 2.39%, respectively, while the proportion of oral health diseases for all ages in Sumedang Regency was 22.40%. 8 This study aims to analyze the relationship between maternal oral health behavior and children's oral health status, especially in Tanjungsari Village, Tanjungsari sub-district, Sumedang, West Java, Indonesia.

## Materials and Methods

### Study Design

The research methodology used is an observational study with a cross-sectional approach to determine the oral health behavior of mothers and children's oral health status. The sampling method and technique used nonrandom and consecutive sampling from six Posyandu in Tanjungsari Village, Tanjungsari District, Sumedang. The research variables were the mother's oral health behavior and the child's oral health status.

### Population and Sample

The population is mothers with children aged between 3 and 5 years in Tanjungsari Village, Tanjungsari District, Sumedang, West Java, Indonesia. If the mother has more than one child aged between 3 and 5 years, the child included in the sample criteria is the first child. The study sample did not include pregnant women and children with special needs. The population in this study was 45 mothers, and based on Slovin's formula, 9 the minimum sample size with a margin of error of 2% and a 95% confidence level was 45 people.

### Development of Measuring Tools

The instruments used in this study were two modified questionnaires from World Oral Health(WHO) 10 that had been translated using the back-forward translation method regarding oral health behavior and oral health status of children to be filled in by each child's mother. The questionnaire used has been translated by the Unpad Language Center and reviewed by experts. The questionnaire was tested on 30 mothers to get the value of validity and reliability. The group of mothers for this trial filled out the questionnaire twice in 2 weeks to assess test–retest reliability. These 30 mothers were not included in the main study. In the follow-up questionnaire, global rankings were replaced with global transition ratings (i.e., questions asking whether the child's oral health had changed since recruitment). This information is needed to calculate the retest–test reliability coefficient as a proportion of the variance in scores attributable to significant differences between patients with stable health status over time. 11 The primary and advanced questionnaires were conducted individually. The scale Cronbach's alpha was 0.88, and the intraclass correlation coefficient was 0.75, indicating substantial agreement.

Examples of questions in this questionnaire include how do you think your child's teeth are. The answer choices are perfect (score 1), very good (score 2), good (score 3), average (score 4), poor (score 5), very bad (score 6), and don't know (score 9). Another question “how often does your child complain of toothache?” and the answer choices are often (score 1), occasionally (score 2), seldom (score 3), never (score 4), and don't know (score 9).

### Data Analysis

The child's oral health status is assessed based on the mother's knowledge and feelings about the child's oral health condition. Analysis of the data in this study used regression and correlation analysis by conducting statistical tests of chi-squared validity and Spearman rank correlation using the SPSS application.

### Research Ethical Aspects

This research has received approval from the Research Ethics Commission of Padjadjaran University Bandung with ethics number 446/UN6.KEP/EC/2022.

## Result

The characteristics of the 46 mothers studied are shown in Table 1 ; the highest proportion is mothers aged between 26 and 30 years (58.7%), and the least is more than 35 years (8.7%). The average age of the mothers studied was 29.3 years. The most recent level of education for mothers is the senior high school level (50%), but there are still elementary school levels (4.3%). Characteristics of children in the 4-year age group showed the most 28 children.

**Table 1:** Characteristics of mother and child

Characteristics	*n*	%
1. Mother's age (years):		
≤ 25	5	10.9
26–30	27	58.7
31–35	10	21.7
> 35	4	8.7
Mean (SD): 29.3 (4.1) Range: 21–42		
2. Mothe's education:		
Elementary school	2	4.3
Junior high school	8	17.4
Senior high school	23	50.0
University	13	28.3
3. Child's age (years):		
3	9	19.6
4	28	60.9
5	9	19.6
4. Child's gender:		
Boys	22	47.8
Girls	24	52.2

Abbreviation: SD, standard deviation.

Table 2 shows the oral health behavior of mothers and children. Mother's oral health behavior found that 84.8% brushed their teeth more than twice daily, and 15.2% brushed their teeth once daily. All mothers who participated in the study used a toothbrush and toothpaste to maintain oral hygiene, but only 41.3% of the mothers studied knew and used fluoride toothpaste.

**Table 2:** Mother and child oral health behavior

Mother's variable	*n*	%	Child's variable	*n*	%
1. Frequency of brushing teeth:			1. Frequency of brushing teeth:		
Never	0	–	Never	0	0
Once a month	0	–	Several times a month	0	0
2–3 times a month	0	–	Once a week	0	0
Once a week	0	–	Several times a week	1	2.2
2–6 times a week	0	–	Once a day	15	32.6
Once a day	7	15.2	More than 2 times a day	30	65.2
More than 2 times a day	39	84.8			
2. Instrument use:			2. Instrument use:		
Toothbrush	46	100	Toothbrush	46	100
Wooden toothpick	20	43.5	Wooden toothpick	1	2.2
Plastic toothpick	0	–	Plastic toothpick	0	–
Dental floss	3	6.5	Dental floss	0	–
Siwak	6	13.0	Siwak	3	6.5
Other tools	0	–	Other tools	0	0
3. Toothpaste usage:			3. Toothpaste usage:		
Yes	46	100.0	Yes	46	100.0
Yes, and with fluoride	19	41.3	Yes, and with fluoride	22	47.8
4. Dentist visit			4. Frequency of dental visit in the last year:		
< 6 months ago	5	10.9	Once	5	10.9
6–12 months ago	4	8.7	Twice	2	4.3
> 1– < 2 years ago	5	10.9	Three times	2	4.3
2–5 years ago	6	13.0	Never been to a dentist	37	80.4
> 5 years ago	11	23.9			
Never been to the dentist	15	32.6			
5. Reason visiting dentist			5. Reason for visiting a dentist		
Consult	7	15.2	Dental pain	3	6.5
Dental pain	19	41.3	Filling/ extraction	1	2.2
Dental treatment	0	–	Routine dental checks up	5	10.9
Routine checks up	5	10.8	Forget		
Don't know/forget	0	–			

Table 3 shows the frequency of maternal dental and oral health problems in the last year. As many as 78.04% of mothers have never experienced dental and oral health problems in the last year. Table 3 shows frequency of mother's food and drink consumption with visible indicators such as consumption of fresh fruits, biscuits, sponge cakes, tarts, sweet bread or pies, jams or honey, chewing gum containing sugar, candy, and soda drinks or sweet packaging, sweet tea, and sweet coffee. Mothers' feelings about their children's oral health are presented in Table 3 , with 17 of 46 mothers feeling that their children's teeth were in good condition and 32 of 46 mothers feeling that their children's gums were in good condition.

**Table 3:** Response to the questionnaire

Frequency of maternal dental and oral health problems in the last 1 year					
Indicator	Response				
	Frequently	Sometimes	Rarely	Never	Not sure
1. Difficulty biting food	1	2	10	32	1
2. Difficulty chewing food	1	2	10	33	–
3. Difficulty speaking/pronouncing some words	–	–	2	44	–
4. Mouth feels dry	–	2	16	27	1
5. Feeling embarrassed about the appearance of your teeth	1	4	9	31	1
6. Avoid smiling because of teeth	1	2	6	36	1
7. Sleep disturbance	–	–	9	36	1
8. Not coming to work	–	–	4	41	1
9. Difficulty with normal activities	–	–	6	40	–
10. Reduce social activities	–	–	5	39	2
Total	4	12	77	359	8
Percentage	0.87	2.61	16.74	78.04	1.74

**Table:** **Frequency of mother's food and drink consumption**

Food and beverage consumption	Response					
	Never	Few times a month	Once a week	Several times a week	Once a day	Two or more in a day
1. Fresh fruits	2	4	4	28	5	3
2. Biscuits. muffin. tart	2	8	1	21	13	1
3. Sweet bread or pie	9	4	3	22	8	–
4. Jam or honey	20	12	5	7	2	–
5. Chewing gum contains sugar	34	6		6	–	–
6. Candy	24	6	–	11	3	2
7. Soft drinks or sweet packaging	17	7	3	11	4	4
8. Sweet tea	21	8	1	10	4	2
9. Sweet coffee	25	3	5	9	4	–
Total	154	58	22	125	43	12
Percentage	37.20	14.01	5.31	30.19	10.39	2.90

**Table:** **Frequency of child's food and drink consumption**

Food and beverage consumption	Response					
	Never	Few times a month	Once a week	Several times a week	Once a day	Two or more in a day
1. Fresh fruits	1	1	7	24	8	5
2. Biscuits. muffin. Tart	–	3	–	22	19	2
3. Sweet bread or pie	13	12	2	11	6	2
4. Jam or honey	14	15	4	12	1	–
5. Chewing gum contains sugar	15	3	2	21	2	3
6. Candy	4	6	3	17	11	5
7. Soft drinks or sweet packaging	1	1	1	16	21	6
8. Sweet tea	15	10	–	15	3	3
Total	63	51	19	138	71	26
Percentage	17.12	13.86	5.16	37.50	19.29	7.06

**Table:** 

Mother's smoking habit	*n*	%
Daily smokers	2	4.3
Non-smoker	44	95.7

**Table:** **Mother's feelings about the child's oral health condition**

Child's oral health status	Mother's feeling						
	Perfect	Very good	Good	Fair	Poor	Very poor	Not sure
Teeth	1	2	17	11	13	1	1
Gum	1	2	32	7	3	–	1

**Table:** **Frequency of complaints of toothache in children in the last 1 year**

*f*	*n*	%
Frequently	3	6.5
Sometimes	8	17.4
Rarely	9	19.6
Never	26	56.5

**Table:** 

Mother's feelings about the condition of the child's teeth	Response		
	Yes	No	Not sure
1. Not satisfied with the appearance of the child's teeth	15	27	4
2. The child avoids smiling or laughing because of the condition of the teeth	1	45	–
3. Other children laugh at the condition of the child's teeth	3	43	–
4. Children don't go to school because of toothache	–	46	–
5. Children find it difficult to bite hard food	12	33	1
6. Children have difficulty chewing	2	44	–
Total	33 (11.96%)	238 (86.23%)	5 (1.81)

Table 3 shows frequency of children's food and beverage consumption, such as fresh fruits, biscuits, sponge cakes, tarts, sweet bread or pies, soft drinks or sweet packaging, jams or honey, chewing sugary gum, candy, sweet milk, and tea sweet. From the results of filling out the survey through a questionnaire, a score was calculated for each aspect of the mother's oral health behavior and the child's oral health status. Scores were calculated by adding the scores of each item of statements and questions asked. The results of the calculation of scores for maternal oral health behavior and children's oral health status are presented in Tables 4 and 5 .

**Table 4:** Statistical description for maternal oral health behavior scores

Subvariable	Statistical size		
	Mean	Median	Range
1. Mother's tooth brushing score	6.85	7	6–7
2. Maternal oral hygiene score	1.65	2	1–4
3. Mother's toothbrush usage score	1.41	1	1–2
4. Maternal verbal health care score	1.72	1	0–5
5. Score the frequency of maternal oral health problems	37.54	39	28–40
6. Score the frequency of mother's food and drink consumption	24.41	24	11–41
7. Mother's smoking habit score	2	2	1–2
Combined	75.54	74.5	61–91

**Table 5:** Statistical descriptions for children's oral health status scores

Subvariable	Statistical size		
	Mean	Median	Percentage
1. Score of mother's feelings about child's oral health	7.24	7.0	3–16
2. Score of the frequency of children's toothache complaints	3.26	4	1–4
3. Child oral health care frequency score	0.33	0	0–3
4. Score pf the frequency of children brushing their teeth	1.72	0	0–5
5. Score of children's instrument use frequency	1.09	1	1–2
6. Score of children's toothpaste usage frequency	1.83	2	1–3
7. Score of the condition of the child's teeth	11.07	11.5	8–12
8. Score of the frequency of children's food and drink consumption	27.93	28.5	16–41
Combined (child oral health status score)	54.46	53.5	43–71

**Table 6:** Correlation of children's oral health status scores with various parameters maternal oral health behavior score

Correlation of children's oral health status scores with	Correlation coefficient (r)	*p-* Value
Tooth brushing score	–0.124	0.413
Oral hygiene score	0.245	0.100
Toothbrush usage score	–0.179	0.235
Oral health care score	–0.287	0.053
Oral health problem frequency score	–0.207	0.168
Food and beverage consumption frequency score	0.437	0.002
Smoking habit score	–0.266	0.074
Maternal oral health behavior score (combined)	0.198	0.188

Abbreviation: *r* , Spearman rank.

The results of the Spearman rank correlation statistical test showed that the maternal oral health behavior score's correlation coefficient ( *r* ) was 0.198 with a *p* -value of 0.188. From the calculation of the Spearman rank correlation, it is known that the mother's oral health behavior that affects the child's oral health status is in the food and beverage consumption selection. The analysis to examine the relationship between children's oral health status and maternal oral health behavior consists of several indicators. The maternal oral health behaviour score obtained a correlation coefficient of 0.198, and a *p* -value of 0.188, which indicates the child's oral health status has a negative correlation with tooth brushing scores, oral hygiene, toothbrush use, maternal oral health care, the frequency of maternal oral health problems, and mother's smoking habit. The score for calculating the correlation with the tooth brushing score obtained a correlation coefficient of −0.124 with a *p* -value of 0.413, while the oral hygiene score was obtained at 0.245 with a *p* -value of 0.100. The correlation calculation with the score of using a toothbrush and maternal oral health care was also carried out, which was −0.179 ( *p* = 0.235) and −0.287 ( *p* = 0.053). For the correlation between the frequency of maternal oral health problems and maternal smoking habits, the results were −0.207 with a *p* -value of 0.168 and −0.266 with a *p-* value of 0.074. The correlation calculation shows no relationship between the score of brushing teeth, oral hygiene, toothbrush use, maternal oral health care, the frequency of maternal oral health problems, and maternal smoking habits with the oral health status of children.

## Discussion

Oral health is a component of good health and plays a vital role in life, especially in children. Behavior is one of the crucial factors that can affect the oral health status of individuals or communities. 7 12 13 H.L. Blum as stated by Flemming distinguishes behaviour into three domains: knowledge, attitude, and action. 14 Dental health behavior is the same as H.L. Blum's theory but is related to the concept of healthy toothache and prevention efforts. Behavior and attitudes can be in the form of knowledge accompanied by a tendency to act according to knowledge. 4 14 15 16 17

The role of parenting on the oral health of their children is divided into two aspects. The first aspect is the role of parents in determining diet patterns, brushing teeth, and using fluoride-containing toothpaste in children. The second aspect is the child's behavior during dental treatment, which reflects the child's overall behavior. 18 19 Of the two aspects, the most decisive in seeing how big the role of parents in their oral health is the first aspect, namely how parents determine the type of food to eat, children consume; how to brush their teeth, and the use of toothpaste that children use. 20 21

Child development is related to all changes that occur in children, physically, cognitively, emotionally, and psychosocially. 14 Psychologists classify children aged between 2 and 6 as groups, explorers, or asking age. The child's first social creature is known and is the first place for the child to socialize with the surrounding environment so that the family becomes very influential in the child's development process. Childhood is the beginning of the formation of behavior, so parents are expected to be able to educate their children on maintaining healthy teeth and mouth properly. 7 The ability to brush teeth is taught and emphasized in children throughout the child's life. Children under 5 years cannot maintain good oral hygiene, so parents must brush their children's teeth until they are 6 years old. Good oral health habits in children reflect parental behavior that positively affects children. 3

The dental and oral health status of children depends on the knowledge and behavior of parents because early childhood has not been able to take care of themselves, so the habits that parents apply will affect the hygiene and oral health status of children. 12 Assessment of oral health status in this study is based on the mother's feelings and knowledge about her child's oral health condition. Based on the descriptive analysis of mothers' knowledge about brushing their children's teeth, it was found that most of the knowledge of mothers in brushing their teeth fell into the good category and was dominant. Primary prevention includes prevention before illness, such as brushing teeth twice a day in the morning after breakfast and at night before going to bed, using dental floss for interdental cleaning, and regular visits to the dentist every 6 months. Fluorine in the right dose is helpful for caries prevention; it has various forms such as mouthwash, toothpaste, and others that dentists can use. To maximize the beneficial effects of fluoride in toothpaste, brushing teeth should be done twice a day and rinsing after brushing should be kept to a minimum or not done entirely. 12 13 22 23 24 25 26

Study on the frequency of children's food and beverage consumption showed that most children consume sweet beverage and food several times a week. In a study conducted by Andriani, every little child really likes to eat sweet foods and not only tastes that appeal to small children but also sees the shape and color of the food and drink. Some parents let their children eat sweet foods, even though they know they are very harmful to teeth. Not many parents tell their children to brush their teeth or rinse their mouths with water after eating sweet foods. Although much information has been provided, until now there are still parents who are not aware of their child's dental health. Many of them still think that their children's teeth are not permanent and will eventually fall out and be replaced with permanent teeth. 27 Several studies also support the results of this study, where the role of parenting patterns in determining the type of food and beverage consumption plays a role in determining the oral health of their children. 28 29 30 31

This study indicates that as many as 95.7% of mothers have good behavior in maintaining oral health by not smoking. According to several studies, good knowledge occurs in attitudes and actions that are followed and seen based on dental and oral hygiene and awareness in maintaining and maintaining dental and oral health. 32 33 34 The maternal oral health behavior score was obtained from the combination of the average scores for brushing teeth, oral hygiene, toothbrush use, maternal oral health care, frequency of oral health problems, maternal food and drink consumption, and smoking habits so that the total score obtained was 75.5. The score of the child's oral health status was obtained from the combination of the average score of the mother's perception of the child's oral health, the frequency of dental complaints, the frequency of the child's oral health care, the frequency of the child tooth-brushing activities, the frequency of using dental instruments, the frequency of using toothpaste, the condition of the child's teeth, and dietary consumption; thus, the overall average score of children's oral health status is 54.5.

The frequency of food and beverage consumption with the score of maternal oral health behavior shows a positive correlation, with a correlation coefficient of 0.437 and a *p* -value of 0.002. These results prove that the indicator of oral health behavior affecting children's oral health status is the frequency of food and beverage consumption. The frequency of maternal food and drinks consumption shows a relationship because some food and drinks are provided by the mother at home so that the child consumes what the mother offers and what mother consumes at home. On the other hand, other behavioral indicators do not show a relationship because some mothers have implemented the proper behavior in maintaining their oral health but did not apply it to their children, or vice versa, mothers used the appropriate behavior in supporting their children's oral health but did not apply it to themselves. Ajzen stated that three psychological determinants of intentional behavior generally affect behavior in the theory of planned behavior. 35 36 These determinants are attitudes related to the evaluation of an individual's habits, subjective norms that refer to consideration of what other people think is essential and beliefs about what must be done, and control of behavioral perceptions that determine the simplicity or complexity of a person's perception of behavior. Attitude towards behavior is an important point that can predict an action but still considers a person's attitude in testing subjective norms and measuring the perceived behavioral control of the individual. For example, suppose a positive attitude is found, support from people around, and the perception of ease because there are no barriers to behavior. In that case, a person's intention to behave will be higher.

Based on the theory put forward by Ajzen, the researcher assumes that children's behavior is due to the attitude carried out after evaluating the habits children see from their parents, especially mothers. This behavior is because the child considers his mother to be an essential and trustworthy person to imitate his behavior. Besides, the researcher assumes that there is control over the perception of behavior from the mother, such as providing facilities or teaching children to behave so that children have high intentions to behave like mother. In this study, the behavior highly imitated by children is the behavior in food and beverage consumption that affects the child's oral health status. 35

The results of this study are supported by previous research by Athavale et al, who stated that parents should know the importance of brushing their teeth and consuming sweet foods for oral health. Consumption of foods that contains carbohydrates too much can increase the potential for caries formation because tooth enamel does not have time to remineralise. 37 Abduljalil et al also stated that a mother's knowledge regarding the oral health of preschool children is considered good. However, it is unfortunate that this knowledge is not fully applied in daily life. Early childhood depends on parents, especially mothers, who act as role models in shaping children's behavior. Children's oral hygiene is essential and based on parental knowledge. Parental knowledge and positive behavior towards good oral care are fundamental in the prevention cycle. 31 The limitation of this study is the location of the study that focuses on one village in Tanjungsari because the situation is still a pandemic. The focus of the research location makes the results less variable.

## Conclusion

Based on all indicators of maternal oral health behavior studied, the behavior in maternal food and beverage consumption has a relationship with the child's oral health status, namely, the better the behavior in the mother's food and drink consumption, the better the child's oral health status. However, other indicators of oral health behavior did not show any relationship with the oral health status of children. It is highly suggested that this study be extended to a wider area with more complex variables to obtain results that can represent a more general situation.
